# Electrical dataset of household appliances in operation in an apartment

**DOI:** 10.1016/j.dib.2023.109742

**Published:** 2023-10-31

**Authors:** Edwin Garabitos Lara, Alexander Vallejo Díaz, Carlos Napoleón Pereyra Mariñez

**Affiliations:** a Faculty of Engineering, Instituto Especializado de Estudios Superiores Loyola (IEESL), 91000 San Cristóbal, Dominican Republic; b Faculty of Science, Escuela de Física, Universidad Autónoma de Santo Domingo (UASD), 10103 Santo Domingo, Dominican Republic; c Faculty of Science, Escuela de Matemáticas, Universidad Autónoma de Santo Domingo (UASD), 10103 Santo Domingo, Dominican Republic

**Keywords:** Electric network analyzer, Electric power profile, Power factor, Energy consumed

## Abstract

This data article presents electricity demand data on common household appliances in the Dominican Republic and therefore in the Caribbean region, as well as appliances whose demand characteristics are maintained regardless of geographic location. A one-hour record is made in most cases, for 17 household appliances, containing information on power demand, effective current, power factor and harmonic distortion of voltage and current. The average, minimum, and maximum values ​​of each parameter for one minute are provided. The data were obtained using the Fluke-1738 electrical network analyzer. The data can be used to build demand profiles in the residential or commercial sector, depending on the composition of electrical appliances and the hours of use, dimension photovoltaic systems for self-consumption and emergency electrical sources. Although the number of household appliances and conditions of use of some appliances do not constitute a representative sample, the dataset and the measurement method constitute a starting point for obtaining this type of records, which are scarce in the literature.

Specifications Table

**Table:** 

Subject	Energy Engineering and Power Technology
Specific subject area	household appliance power demand profile and power factor
Data format	Raw Filtered Change of format and specifications
Type of data	FCA2 files CSV files xlsx files Images
Data collection	Data from July 8th 2023 to August 22nd 2023: ­ Report of the plate data of the electrical household appliances. ­ Records of energy demand profiles with a resolution of one minute and in some cases by seconds, for a period of one hour in most cases. ­ Record of maximum power demand for each household appliance, energy consumed and power factor.
Data source location	Institution: Instituto Especializado de Estudios Superiores Loyola (IEESL). City/: San Cristóbal. Country: Dominican Republic. Latitude and longitude for collected data: Lat.: 18.41, Long.: 70.10.
Data accessibility	Repository name: *Electrical dataset of household appliances in operation in one apartment*[1] Data identification number: 10.17632/vn2cks9vdr.2 Direct URL to data: https://data.mendeley.com/datasets/vn2cks9vdr/2

## 1 Value of the Data

• The data set provides the power profile and others electrical parameters of the most common household appliances in a middle-class family in the Dominican Republic. This information does not appear on the nameplate of the appliances and is scarce in the literature.
• The joint demand profile of a home is usually analyzed and used, but not individually for each appliance. Individual profiles allow for a better understanding of the electrical behavior of different appliances according to the time and the conditions of use.
• The dataset can be mainly useful for electrical engineers, power demand planners, inverters sizing, emergency generators, other low voltage engineering applications or carry out research.
• Researchers can use the dataset to estimate energy demand based on the electrical appliances used and thus the cost of the household bill, estimation of the power factor according to the type of household appliances, the sizing of photovoltaic self-consumption and other research.

## 2 Background

The demand for electrical energy is increasing and the residential sector represents approximately 22 % of this [2]. The increase in the participation of renewable energies makes the operation of power systems more unstable and expensive. This has motivated the study of demand response management, including in the residential sector, as a measure to match generation with demand [3]. From the point of view of residential photovoltaic self-consumption, it is necessary to know the energy demand profile for the sizing of photovoltaic systems that operate without surpluses [4]. The data set presented in this work is intended for the prediction, design and construction of demand profiles based on individual demand dataset for household appliances. This information can be used to predict the energy consumed in correspondence with the use of certain appliances and the time of use, as well as the power demanded, information that can be used for energy planning, sizing emergency generation systems, electrical inverters, residential photovoltaic self-consumption and other low voltage applications.

## 3 Data Description

The measurements were carried out in an apartment in San Cristóbal, province of the Dominican Republic. A middle-class family lives in the apartment, consisting of two adults, two children and a teenager; a domestic worker usually stays in the apartment during the day, Monday through Friday. A criterion for choosing household appliances was not applied, but rather the availability of the electrical network analyzer and access to the apartment of one of the authors was taken advantage of.

It should be noted that the measurements were carried out with the appliances in natural operation (protecting the instrument and taking electrical safety measures), except for the compact fluorescent lamps, which, being connected to the ceiling, had to be connected to an experimental socket that allowed the use of the measuring instrument.

Most equipment has a little variable power demand, so that its profile and electrical energy consumption can be scaled to a longer or shorter time, starting from one hour of measurement with a resolution of one minute. Within this group of appliances are laptops, compact fluorescent lamps, wall fans, televisions, television boxes and routers. Due to the variability in their power demand, other appliances require a measurement interval greater or less than one hour. For refrigerators, a measurement time of 24 h was considered for one case and 4 h for another (due to some limitations). For air conditioning, a measurement interval equal to that of its usual use was considered. In the case of appliances that are rarely used per week, such as washing machines and irons, some minutes were considered with a resolution of seconds, in order to capture the variations. The appliances and measurement context are listed in Table 1 so that the data sets can be used reasonably.

**Table 1:** List of appliances and measurement context.

#	Type	File name	Measurement time	Resolution	Measurement context
1	Air conditioner	AA1	8 h 5 min 36 s	1 min	The room has dimensions of 5.40 m x 3.30 m x 2.63 m and sleeps two adults. The outdoor side and the indoor temperature were 28 °C and 22 °C, respectively. The equipment is usually only used when going to bed.
2	Lightbulb	CFL1	1 h 0 min 0 s	1 min	The Light bulb had to be plugged into an experimental socket in to use the Fluke analyzer.
3	Electric iron	Electric iron	22 min 0 s	1 s	Only a couple of clothes were ironed, this is why the measurement time is short.
4	FAN WALL	FAN WALL1	1 h 0 min 0 s	1 min	The fan is used at the lowest speed of three that have.
5	FRIDGE	FRIDGE1	1 d 0 h 0 min 0 s	1 min	Normal usage context.
6	FRIDGE	FRIDGE2	2 h 53 min 37 s	1 min	This measurement was carried out in a other place different to the apartment and it was not possible to measure for one day.
7	LAPTOP	LAPTOP1-hp	1 h 0 min 0 s	1 min	The laptop was used during the measurement.
8	Monitor	Monitor-24SAMSUNG	1 h 0 min 0 s	1 min	The Monitor was used during the measurement.
9	Monitor	Monitor2–24DELL	1 h 0 min 0 s	1 min	The monitor was used during the measurement.
10	Router	Router ALTICE	1 h 0 min 0 s	1 min	This device is always in operation.
11	TV	TV1–32OFF	1 h 0 min 0 s	1 min	Demand is measured when the TV is turned off.
12	TV	TV1–32ON	1 h 0 min 0 s	1 min	Demand is measured when the TV is on and in use.
13	TV	TV-50ON	1 h 0 min 0 s	1 min	Demand is measured when the TV-50ON is turned on and in use.
14	TV-BOX	TVBOX1-OFF	1 h 0 min 0 s	1 min	Demand is measured when the TVBOX1 is turned off; this device is always in operation.
15	TV-BOX	TVBOX1-ON	1 h 0 min 0 s	1 min	Demand is measured when the TVBOX1 is turned on; this device is always in operation.
16	Washing machine	Washing machine	20 min 13 s	1 s	The operating time of the device was only 20 min, a very limited time for the behavior of this appliance.
17	WIFI repeater	WIFI repeater	1 h 0 min 0 s	1 min	This device is always in operation.

The information consists of three data sets, which contain energy demand profiles of different household appliances as well as parameter information on energy quality, obtained with the Fluke-1738 electrical network analyzer. The three data sets are described below.

1. Raw files-Appliance data: These are 17 FCA2 files that are obtained from the Fluke-1738 and processed in the “Fluke Energy Plus 3.10 software”.
2. Filtered CSV files - Appliance Data: There are 17 CSV files that are exported from the 17 FCA2 files, but in this case, they contain only the most relevant information.
3. Filtered xlsx files and specifications-Appliance Data: There are 17 xlsx files that are exported to Excel spreadsheets from the 17 CSV files and that additionally contain information about the plate data of each appliance, in an additional Excel spreadsheet. This data set is the final product and the reason for this documentation. Table 2 indicates the concepts and description of the information contained in the two spreadsheets within each file.

**Table 2:** Description of the two sheets in each xlsx file in the folder “Filtered xlsx files and specifications”.

**Sheet 1: Appliance name _Description**	
**Concept**	**Description**
Plate data	It refers to the plate data on each appliance, such as brand, model, voltage, power and nominal current.
Additional information	It refers to information that can influence the energy demand of the electrical appliance, so it must be indicated.
Measuring instrument	It refers to the instrument used to measure, the measurement time, the resolution, the start time and date, and the end time and date.
Images	It refers to images of the physical appliance, where the plate data is made visible.

**Sheet 2: Appliance name _Data**	

**Concept**	**Description**
Start (SA Western Standard Time)	Refers to the time at which the measurement begins.
Stop (SA Western Standard Time)	Refers to the time at which the measurement ends.
Trend_Period	Refers to the time resolution in seconds configured for measurements in the electrical network analyzer.
Voltage	It consists of three columns, with the average, minimum and maximum rms voltage in volts (V) over the chosen resolution interval, usually 1 min.
Current	It consists of three columns, with the average, minimum, and maximum rms current over the interval of the chosen resolution interval.
Voltage THD	It consists of three columns, with the average, minimum, and maximum total harmonic distortion (THD) voltage in volts (V) over the chosen resolution interval.
Current THD	It consists of three columns, with the average, minimum and maximum total THD current in amperes (A) over the chosen resolution interval.
Frequency	It consists of three columns, with the average, minimum and maximum values ​​of the fundamental frequency in Hz during the interval of the chosen resolution.
Active power	It includes six columns of data: • Three columns with the average, minimum and maximum values of the active power in Watts (W) during the chosen resolution interval. • Three columns with the average, minimum and maximum values of the fundamental active power in W during the chosen resolution interval.
Non-Active power	It includes six columns of data: • Three columns, with the average, minimum and maximum values of the non-active power in var during the interval of the chosen resolution, usually 1 min records. • Three columns with the average, minimum and maximum values of the fundamental non-active power in var during the chosen resolution interval.
Apparent Power	It includes six columns of data: • Three columns with the average, minimum and maximum values of the apparent power in VA during the chosen resolution interval. • Three columns with the average, minimum and maximum values of the fundamental apparent power in VA during the chosen resolution interval.
Power Factor	It includes six columns of data: • Three columns, with the average, minimum and maximum values of the power factor during the chosen resolution interval. • Three columns, with the average, minimum and maximum values of the displacement power factor during the chosen resolution interval.

**Fig. 1:**
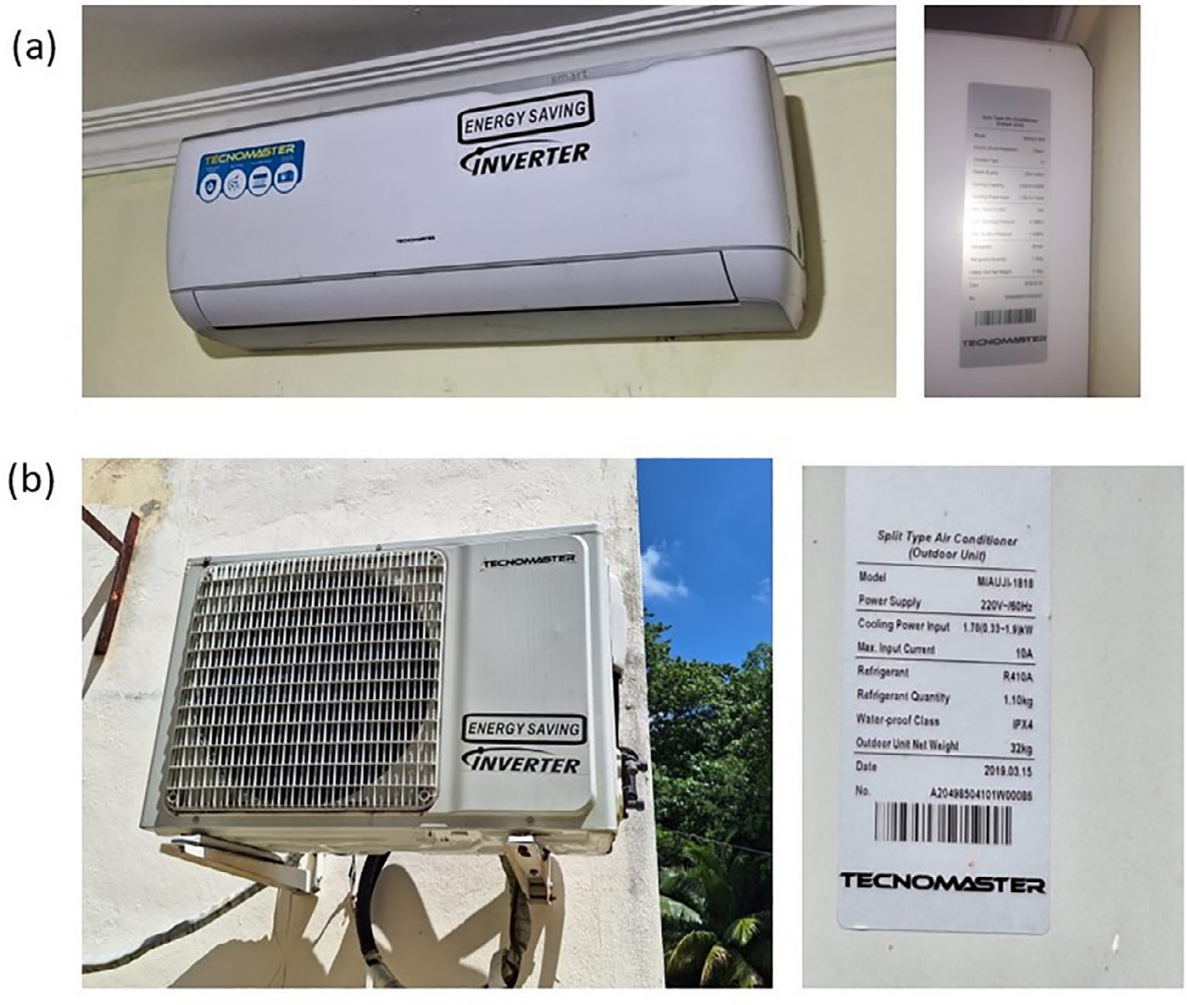
Images of AA1 air conditioner. (a) Image and data of the internal unit (evaporator) and (b) Image and data of the external unit (condenser).

An example of an air conditioner profile is shown in Table 3, showing the content of the first Excel spreadsheet. In some cases, a Note is placed on information that may influence the energy demand of the appliance, in this case, the dimensions of the room and the number of people sleeping are indicated. Fig. 1 shows the images of this air conditioner.

**Table 3:** Equipment datasheet and registration information.

**Place data**	
Type	Air conditioner
Name	AA1
Brand:	TECNOMASTER
Model:	M/AUJI-1818
Nominal voltage (VAC):	220.00
Frequency (Hz)	60
Max. input current (A):	10.0
Capacity (BTU):	18,000
Outside temperature (Celsius grade)	28
Indoor temperature (Celsius grade)	22
**Measure instrument**	
Brand and model	Fluke-1738
Measurement time	8 h 5 min 36 s
Resolution time	1 min
Start	10/7/2023, 11:01:34 PM
End	11/7/2023, 7:07:10 AM
Note:	The room has dimensions of 5.40 m x 3.30 m x 2.63 m and sleeps two adults.

**Fig. 2:**
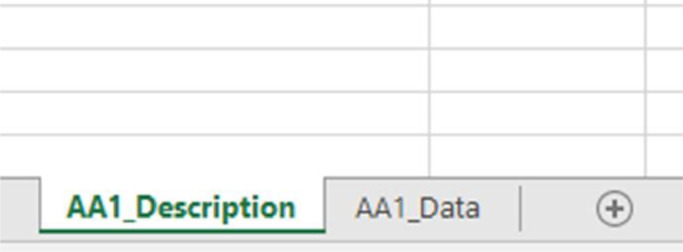
Image of the Excel spreadsheets, to show what they look like, the first is for “Description” and the second for “Data”.

Table 4 shows a fraction of the data contained in the second Excel spreadsheet, which is contained in the spreadsheet named “AA1_Data”, while the descriptive data of the equipment is found in the spreadsheet named “AA1_Descrption”, as shown in the image of Fig. 2.

**Table 4:** Fraction of the measured data of air conditioner AA1 with the power network analyzer.

Start (SA Western Standard Time)	Stop (SA Western Standard Time)	Trend_Period	Vrms_AN_avg	Vrms_AN_min	Vrms_AN_max	→
01:34.2	02:00.0	60	241.049	240.544	241.489	→
02:00.0	03:00.0	60	240.134	239.554	240.808	→
03:00.0	04:00.0	60	239.938	238.997	240.397	→
04:00.0	05:00.0	60	239.578	239.038	240.08	→
05:00.0	06:00.2	60	239.706	239.227	239.975	→
06:00.2	07:00.2	60	239.843	239.348	240.271	→
07:00.2	08:00.2	60	240.231	239.881	240.501	→
08:00.2	09:00.1	60	240.087	239.582	240.428	→
09:00.1	10:00.1	60	240.366	239.918	240.586	→
10:00.1	11:00.1	60	240.357	239.417	240.71	→
↓	↓	↓	↓	↓	↓

## 4 Experimental Design, Materials and Methods

Is remembered that the data and measurements have been collected in an apartment in the Dominican Republic, in the province of San Cristóbal (Lat, Lng: 18.41, −70.10). The measurements were carried out with the Fluke 1738 electrical network analyzer.

### 4.1 Description of measurement and configuration equipment

**Fig. 3:**
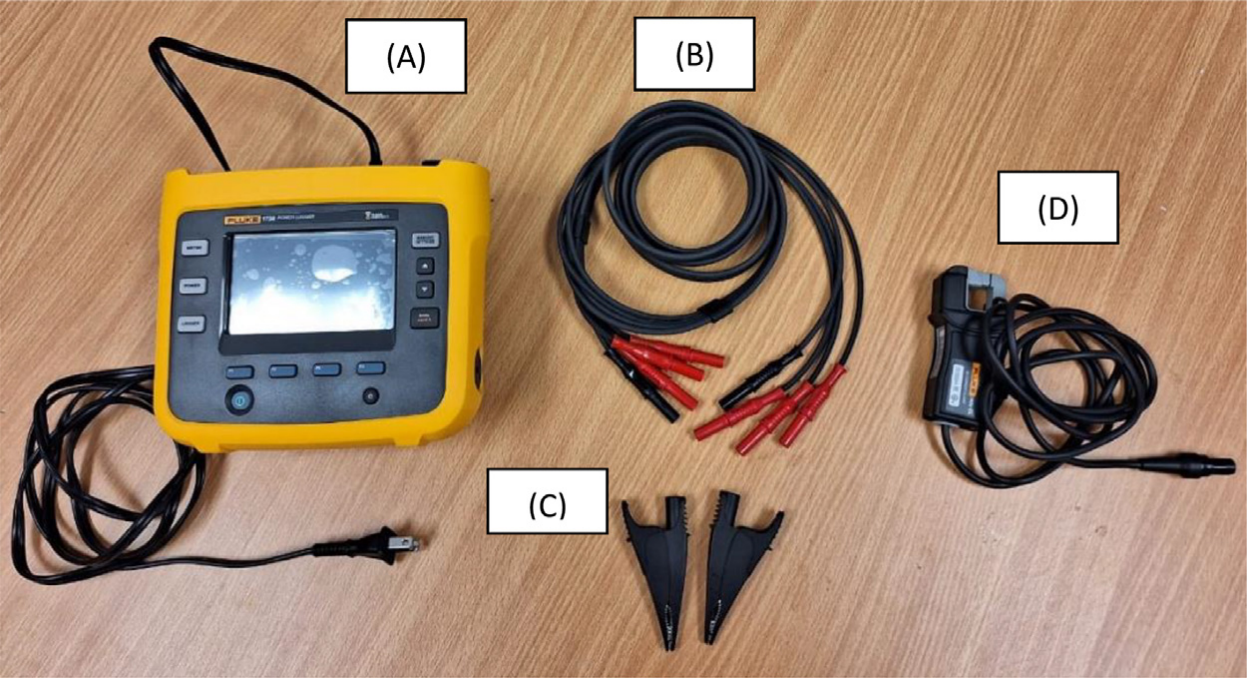
(A) Fluke-1738 electric network analyzer with AC power connector. (B) 4-wire cable, neutral N and lines A, B and C. (C) Two alligator clips. (D) Fluke-17xx i40s-EL current clamp.

The Power Logger 1738 is a compact device for conducting energy and power quality studies. It contains an integrated touch screen and a USB Flash port that allows you to easily configure, verify and download measurement sessions carried out at the measurement site itself, without the need for a computer. It has a battery, which allows you to carry out measurements for approximately one hour. For longer measurements, it must be powered from an AC input voltage source from the power cable 100–240 V 50/60 Hz 15 VAC. Fig. 3 shows the standardized elements used to make the measurements.

Below are the steps to configure the equipment and start data logging

Step 1. Turn on the analyzer using the power button and select the METER option.
Step 2. Within the METER option, the following fields are filled with the options shown
Study Type: Energy study
Power quality: EN 50,160
Topology: Single Phase
Nominal voltage: 120 V
Scale factors for external PTs or CTs: 1:1
Nominal Frequency: 60 Hz

**Fig. 4:**
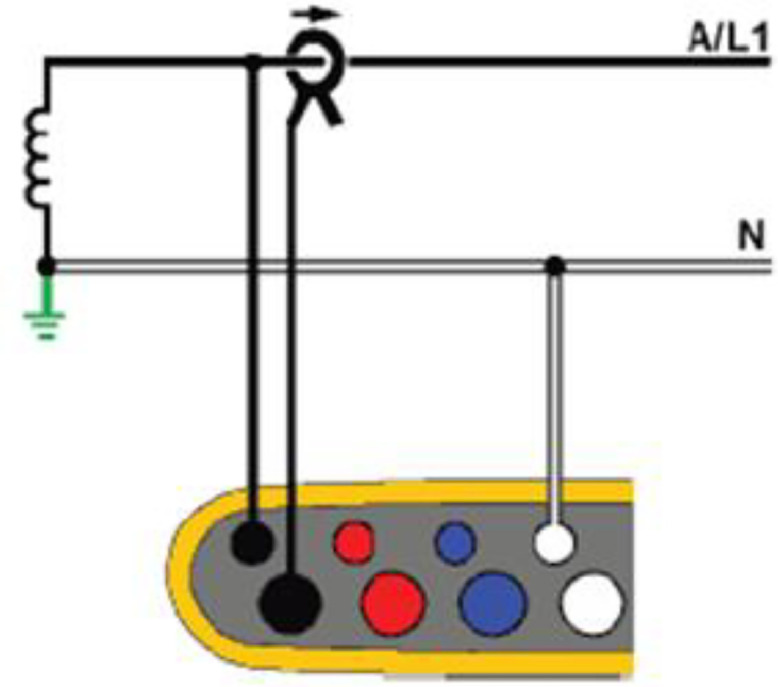
Single phase topology.

The diagram corresponding to the topology must correspond to the diagram shown in Fig. 4, these diagrams appear on the Fluke 1738 screen.

Step 3. Press the LOGGER option, within which the following log fields are filled out and the option to save is given.

Name: Laptop
Duration: 1 h 0 min 0 s
Interval of the average calculation: each 1 min
Demand Interval: 15 min
Energy costs: 0.107 USD/kWh
Description: Profile

Step 4**.** Finally**,** the starts registration option is pressed, and the analyzer begins to measure and record the parameters.

### 4.2 Assembly and electrical connections

**Fig. 5:**
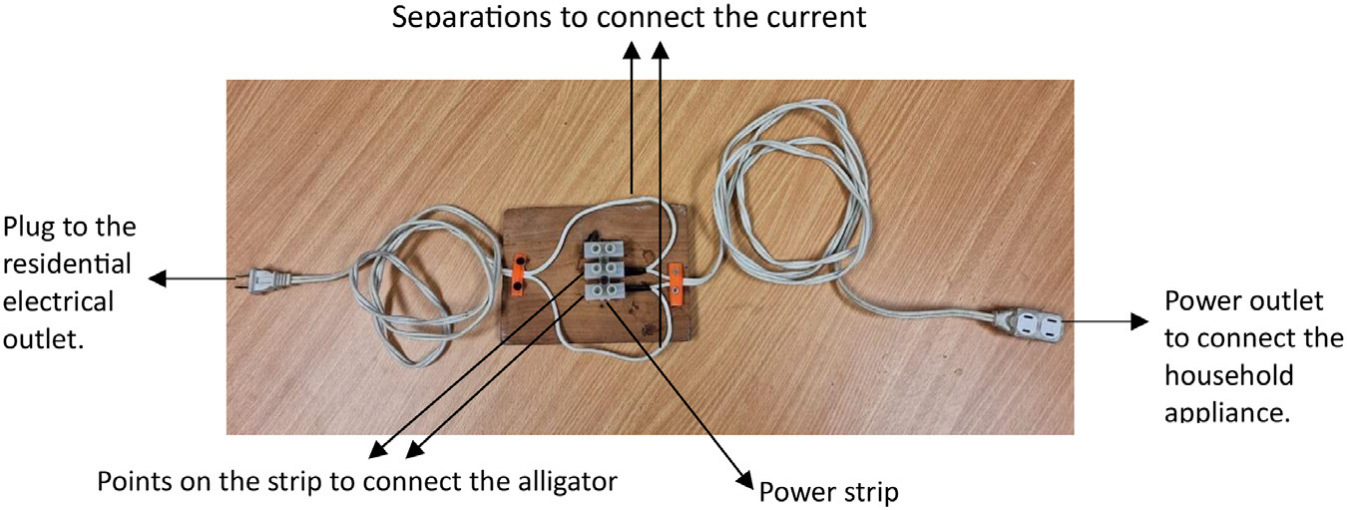
Electric extension to facilitate the connection of the electrical analyzer.

The Fluke 1738 [5] is specially designed to carry out measurements on industrial electrical panels, to measure joint demand of several pieces of equipment or total demand. For this reason, an electrical extension was developed that facilitates the connection of the current clamp and the two voltage clamps, as shown in Fig. 5.

**Fig. 6:**
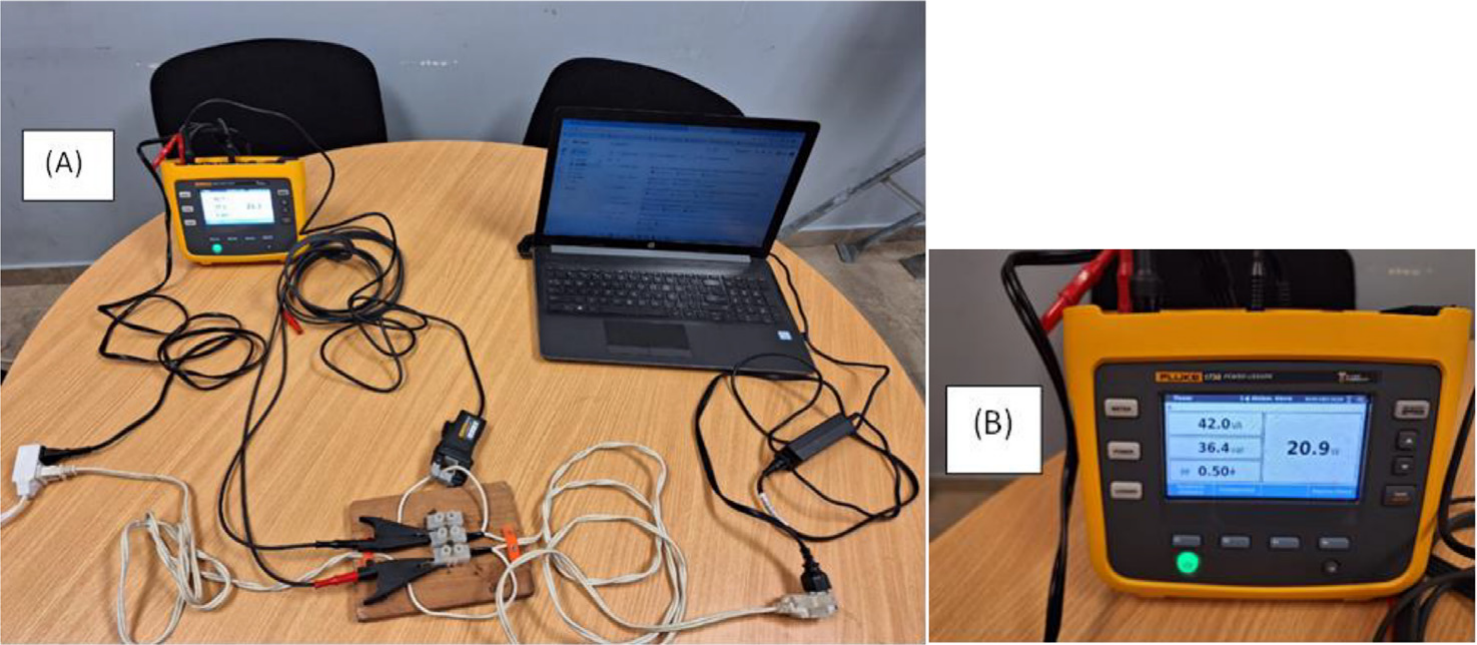
Demonstrative connection to record demand data for a laptop. (A) Demonstrative connection. (B) Power values ​​measured by the electrical network analyzer.

Fig. 6(A) shows a demonstrative connection to measure the demand for a 15-inch laptop. In this case, both the load and the analyzer are powered from the same outlet. For safety, during the implementation, the parts with non-insulated connections were covered, such as the table where the power strip is located, specifically where the alligator clips are connected. Fig. 6(B) shows the screen of the electrical network analyzer in the “Power” option, where the powers and power factor of the laptop are shown.

### 4.3 Data acquisition using Fluke energy analyze plus 3.10 software

**Fig. 7:**
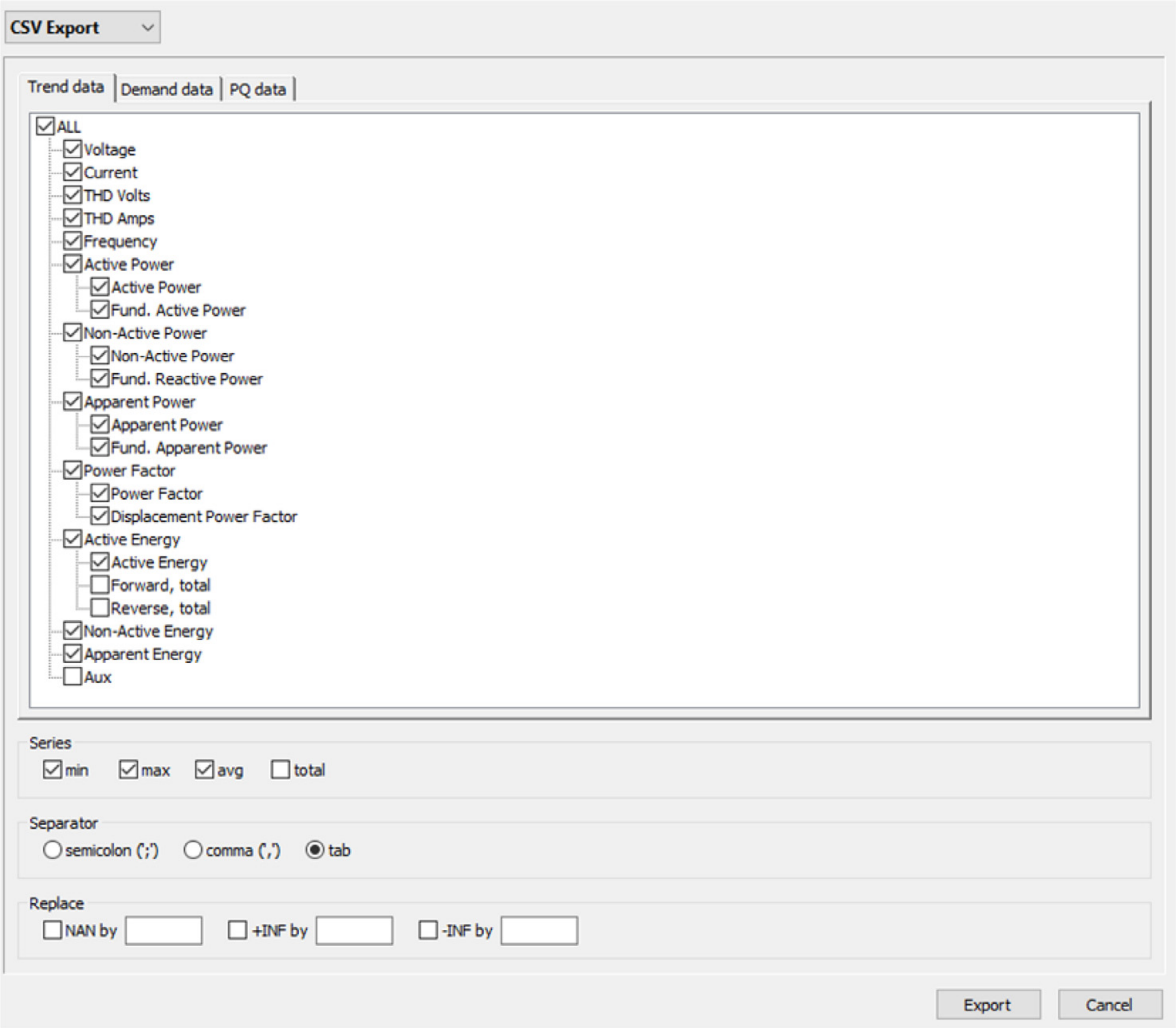
Registry management for download as CSV file.
Step 3. The file is exported to Excel file and forms the second Excel spreadsheet, for example “AA1_Data” in Fig. 2.

After the quantities are measured and recorded in the Fluke 1738, the following procedure was carried out:

Step 1. The log is downloaded through the Fluke 1738′s Wi-Fi or through its USB port. Once you have the log file on a laptop, it is opened using the Fluke Energy Plus 3.10 software. This software recognizes FCA2 Files.
Step 2**.** Select the Project Manager option, the “Export” icon, where the “CSV Export” file type is selected. In the Trend Data option, check the options shown in Fig. 7:

## 5 Limitations

The main limitation in the research was the time available to carry out measurements in the case of some household appliances, as well as the conditions of use. Appliances whose demand profile is very variable require a longer measurement time to be able to make a good scaling in the applications, for example, the washing machine, the electric iron and the fridge. Is a huge variety of appliances available in the market, which means that the number of appliances in this paper is not a representative sample; however, the data set and the measurement method constitute a starting point in obtaining this type of records, which are scarce in the literature.

## Ethics Statement

The data was taken with the consent of the residents. The authors have read and followed the ethical requirements for publication in Data in Brief and confirming that the current work does not involve human subjects, animal experiments, or any data collected from social media platforms.

## CRediT authorship contribution statement

**Edwin Garabitos Lara:** Conceptualization, Methodology, Investigation, Data curation, Writing – original draft. **Alexander Vallejo Díaz:** Conceptualization, Visualization, Writing – review & editing. **Carlos Napoleón Pereyra Mariñez:** Conceptualization, Funding acquisition.

## Declaration of Competing Interest

The authors declare that they have no known competing financial interests or personal relationships that could have appeared to influence the work reported in this paper.

## Data Availability

Electrical dataset of household appliances in operation in one apartment (Original data) (Mendeley Data)
